# Characterization of the complete chloroplast genome of *Rumex dentatus* L. (Polygonaceae)

**DOI:** 10.1080/23802359.2022.2054380

**Published:** 2022-03-28

**Authors:** Suping Zhang, Bingao Guo, Sheqi He, Yanxia He

**Affiliations:** a School of Pharmacy, Henan University, Kaifeng, China; b Institutes of Traditional Chinese Medicine, Henan University, Kaifeng, China; c School of Life Sciences, Henan University, Kaifeng, China

**Keywords:** Chloroplast genome, *Rumex dentatus*, phylogenetic tree

## Abstract

*Rumex dentatus* L. is a flowering plant with promising therapeutic effects. Here, we reported the complete chloroplast genome sequence of *R. dentatus*. The length of the complete genome is 159,103 bp, with a pair of inverted repeat regions (IRb/IRa, 30,628 bp) separated by a large single-copy (LSC, 84,848 bp) region and a small single-copy (SSC, 12,999 bp) region. The overall GC content of the genome was 37.6%, and the corresponding values in the LSC, SSC, and IR regions were 35.8%, 32.6%, and 41.1%, respectively. There are 129 genes, including 84 protein coding genes, 37 tRNA genes, and eight rRNA genes. Phylogenetic tree showed that *R. dentatus* was most relative to the species of *R. nepalensis* and *R. crispus*.

*Rumex dentatus* Linnaeus 1771 is an annual or rarely biennial flowering herb in family Polygonaceae, which is native mostly to east and south Asia (China, India, Japan, and Korea) (Li et al. 2003). As a traditional and folk medicines, *R. dentatus* has a broad spectrum antioxidant and antimicrobial activity, and it may become a potential therapeutic option for many diseases (Humeera et al. 2013). Furthermore, Riffat et al. (2017) have reported that *R. dentatus* plays an important role in inhibiting proliferation and inducing apoptosis of breast cancer MDA-MB-231 cell line *in vitro*. The components of anthraquinone and naphthalene glucosides isolated from *R. dentatus* showed anti-proliferative activity for gastric cancer, breast cancer, melanoma, and oophoroma cell lines (Zhang et al. 2012). It is necessary to study the genetic information of this plant furthermore. With the emergence of high-throughput sequencing technology, it becomes a reality to obtain a large number of genomic date quickly (He et al. 2017). The present study is the first to report the complete chloroplast (cp) genome of *R. dentatus.* The cp genome sequence is deposited in GenBank under the accession number MZ964419.

Healthy and fresh leaves of *R. dentatus* were sampled from the lakeside of the campus of Henan University, China (34°49′12.11″N, 114°18′9.20″E). A voucher specimen (no. HENU20210730) was deposited at the herbarium of School of Pharmacy, Henan University (Kaifeng City, Henan Province; Wangjun Yuan; 10200068@vip.henu.edu.cn). Total genomic DNA was extracted with the modified SDS method. High quality DNA was sheared, and paired-end libraries were constructed, which were sequenced using Illumina NovaSeq PE150 at the Beijing Novogene Bioinformatics Technology Co., Ltd. (Beijing, China). After filtering low-quality reads, approximately 5 Gb clean data were obtained, which was assembled into contigs using a CLC Genomics Workbench 9.5.2 (CLC Inc., Aarhus, Denmark). Then, the complete cp genome was constructed and annotated using Geneious 2020.0.5 (Biomatters, Auckland, New Zealand) following the description of Liu et al. (2017), with the sequence of *R. nepalensis* (GenBank accession no. MT457825) as a reference.

The cp genome of *R. dentatus* was 159,103 bp in length, with a pair of inverted repeat regions (IRb/IRa, 30,628 bp) separated by a large single-copy region (LSC, 84,848 bp) and a small single-copy region (SSC, 12,999 bp). The overall GC content of the genome was 37.6%, and the corresponding values in the LSC, SSC, and IR regions were 35.8%, 32.6%, and 41.1%, respectively. The genome comprised a total of 129 genes: 84 protein-coding genes, eight ribosomal RNA genes, and 37 tRNA genes. Among these genes, nine protein-coding genes (rps16, rpoC1, rpl16, rp12, petD, petB, ndhB, ndhA, atpF) contained one intron, whereas three genes (ycf3, rps12, clpP) contained two introns. Moreover, the rps12 gene was trans-spliced with the 5′ end located in the LSC and the 3′ end duplicated in the IR regions.

The phylogenetic tree, including *R. dentatus* and other 27 species of Polygonales with *Arabis paniculata* as outgroup, was constructed on whole cp genome sequences using the maximum-likelihood (ML) method implemented in RAxMLHPC v8.1.11 on the CIPRES cluster (Stamatakis 2014). The results showed that *R. dentatus* formed a clade with *R. nepalensis* with high bootstrap value (Figure 1). The topology of Caryophyllales is identical to that of the result (Yao et al. 2019). This result will provide valuable insight into conservation and evolutionary histories for this important species.

**Figure 1.**
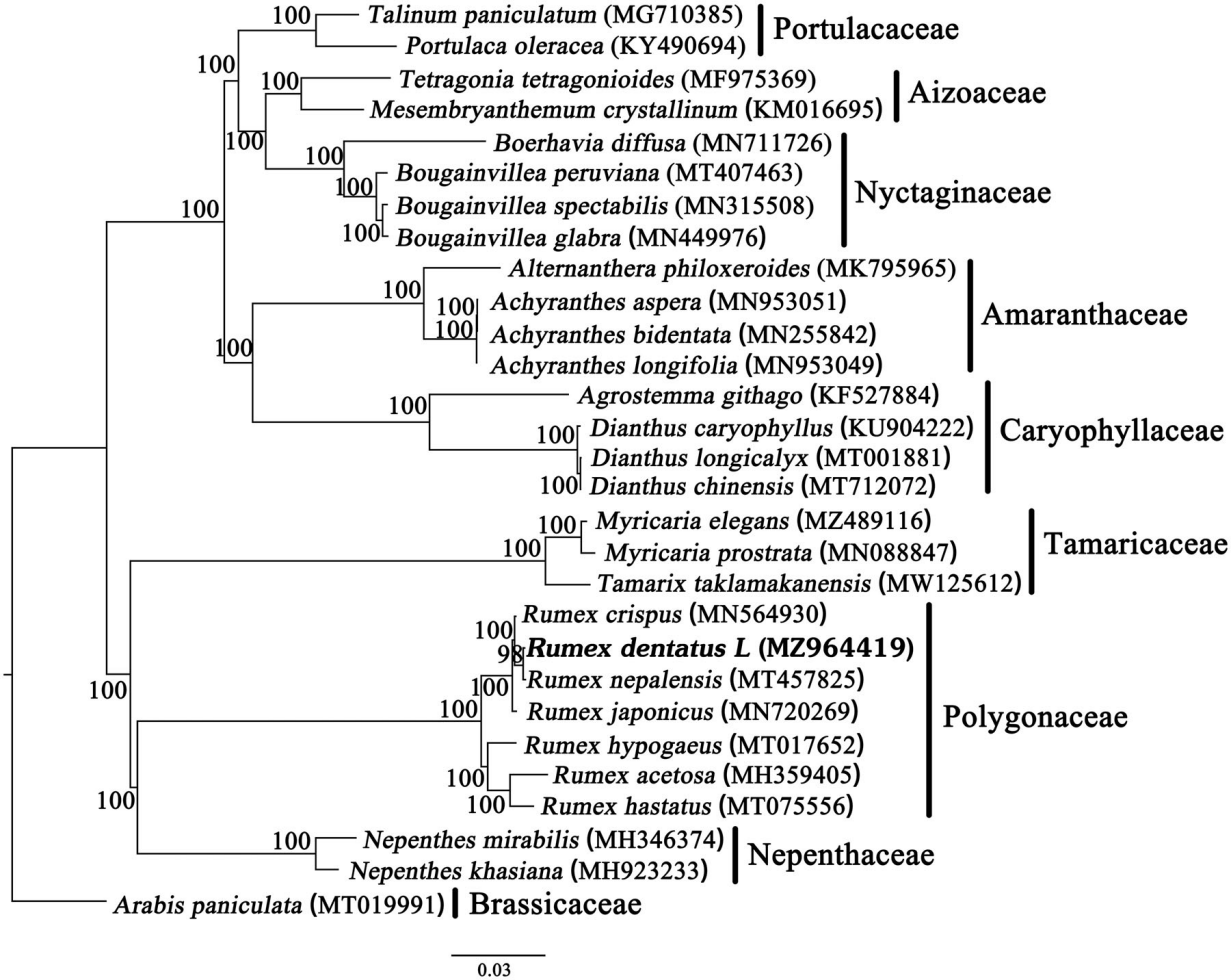
The phylogenetic tree based on 29 chloroplast genomes. Number above each node indicates the ML bootstrap support values.

## Authors contributions

Yanxia He conceived the research project. Suping Zhang, Bingao Guo, and Sheqi He were involved in the analysis of the data. Suping Zhang drafted the paper, and Yanxia He revised it critically for intellectual content. All authors have read and approved the final manuscript. All authors agree to be accountable for all aspects of the work.

## Data Availability

The genome sequence data that support the findings of this study are openly available in GenBank of NCBI at https://www.ncbi.nlm.nih.gov/ under the accession no. MZ964419. The associated BioProject, SRA, and Bio-Sample numbers of the raw sequence data and the genome are PRJNA759788, SRR15698557, and SAMN21197594, respectively.
